# Healing the Healers: Fifty Years of Global Challenges and Progress in Nurse Psychological Wellbeing

**DOI:** 10.1111/jan.70436

**Published:** 2025-12-17

**Authors:** Jill Maben

**Affiliations:** ^1^ School of Health Sciences, Faculty of Health and Medical Sciences, University of Surrey Surrey UK

## 1 Introduction

Global health workforce shortages are projected to reach up to 10 million by 2030 (WHO 2022a), and protecting nurses' psychological wellbeing is essential for attracting and retaining staff who are critical to sustaining current and future healthcare delivery.

Over the past 50 years, the nursing profession has undergone profound transformation, particularly in the area that has shaped much of my own career: nurse psychological wellbeing at work. Having begun my nurse training in 1979, my professional journey has almost spanned the lifetime of this journal, 47 of its 50 years.

When training as an RN (not a degree programme) the focus was on clinical competence, obedience and discipline, with nothing related to the emotional or psychological realities of the work. The curriculum contained little, if anything, about stress, burnout, or moral distress, despite my early and vivid experiences of all three.

Equally in clinical practice there was minimal recognition or support for the psychological demands placed on nurses and we were very much thrown in at the very deep end with students carrying extraordinary responsibilities; being left in charge of a men's surgical ward on night duty as a third‐year student, with only a second‐year colleague alongside me is a stark and very stressful memory. At the time, not knowing what I didn't know felt protective, ignorance as insulation, but it was certainly not protective for the patients. Just 2 years after qualifying, I left nursing; I thought for good, overwhelmed by burnout and moral distress (Maben 2015). My interest in the psychological well‐being of nurses began then, although it would be another 10 years before I embarked on an academic research career in this area.

Historically, nursing emphasised duty, moral resilience and self‐sacrifice (Baly 1995) and the recognition of the importance of nurse wellbeing at work developed slowly. I believe the first paper in the Journal of Advanced Nursing (JAN) to focus on psychological wellbeing was written by Sister Dolan and published in 1987 (Dolan 1987); it examined the relationship between burnout and job satisfaction in nurses. The shift toward exploring psychological aspects of nursing, including stress, burnout and emotional resilience became more prominent in the 1990s, as the profession began to recognise the importance of the psychological health of nurses in relation to nursing practice.

Today, the concept of nurse psychological wellbeing and self‐care has become a cornerstone of professional practice, central to workforce sustainability, the quality and safety of patient care and the performance of entire health systems. In this commentary, I trace this journey and highlight the key work that has shaped our understanding of this fundamentally important topic, including insights from my own research.

## 2 Trends in Nurse Psychological Wellbeing

The work of Isabel Menzies Lyte in 1960 was one of the first studies to examine nurse psychological wellbeing. She took a case‐study approach to the social problems of nursing, identifying that nurses' work generates high levels of anxiety, which organisations manage by creating social systems and routines that may protect nurses collectively, but may displace stress onto individual staff. Her ‘social defenses against anxiety’ theory explains how institutional structures, role fragmentation and ritualised procedures reduce immediate emotional strain while preventing individuals from processing fear, leaving nurses more stressed and less able to respond creatively (Menzies 1960).

A decade later, broader nursing education reform already enacted in many countries, began to shift nurse roles from purely bedside tasks toward more autonomous and expanded practice roles (e.g., in the UK, the Briggs Report of 1972 laid out the need for nurse research, professional development and greater autonomy) (Briggs 1972). These changes indirectly impacted wellbeing by altering role expectations, job design and professional identity.

Studies in the 1970's focused on stress, examining job strain and emotional exhaustion in nursing populations (Freudenberger 1974; Maslach 1976) and although the term burnout was coined in 1974 (Freudenberger 1974), it was in the 1980's that the Maslach Burnout Inventory (MBI) was developed (Maslach and Jackson 1981) which has come to define elements of nurse psychological wellbeing. While initially not nursing‐specific, it was soon applied to nursing and became foundational for research on nurse psychological wellbeing providing the first validated tool to measure the three aspects of burnout: emotional exhaustion, depersonalisation and reduced personal accomplishment.

Research in the USA in the 1980's, started to focus on the environments in which nurses worked. Aiken (1982) examined how the organisation and nurse staffing of hospitals, the ‘work environment’, affected both nurses and patients. The Magnet hospitals research (McClure 1983) identified a small set of hospitals that consistently attracted and retained nurses by creating excellent practice environments. Hospitals with supportive leadership, strong nurse autonomy, opportunities for development and collaborative nurse–physician relationships showed lower turnover, less burnout and better patient outcomes; environments that enabled nurses to exercise control, feel supported and practise in ways that reflected their professional values.

Some of my early research demonstrated how important this alignment with professional values is for nurse retention. In my doctoral work, I identified sustained idealists as those practising in environments that allow new nurses to uphold the values instilled during their education. In contrast, compromised idealists experience an erosion of these values through organisational pressures. This leads to moral distress, lower job satisfaction and partial withdrawal from aspects of care. Finally, crushed idealists have their initial values overwhelmed by systemic constraints. They experienced profound disillusionment and emotional exhaustion and left the profession early, some after as little as 15 months post‐qualification (Maben et al. 2006, 2007).

Furthermore, over time there has been increasing attention paid toward moral distress (Jameton 1984; Wilkinson 1987). This occurs when actions conflict with deeply held values. Psychological distress, frustration and a sense of compromised integrity follow when external constraints, institutional policy, resource shortages or hierarchical decisions prevent someone from acting on their own moral values or ethical judgement. We have also seen growing recognition of vicarious trauma and secondary traumatic stress among frontline staff (McCann and Pearlman 1990; Figley 1995) which have been applied to nursing work more recently (e.g., Sinclair and Hamill 2007; Nolte et al. 2017).

The landmark US report *To Err Is Human* (Institute of Medicine 1999) also highlighted systemic contributors to adverse outcomes, linking staff wellbeing to patient safety. In 2014 the US ‘Triple Aim’– improving the patient experience of care, improving the health of populations and reducing per capita cost, was expanded to the ‘Quadruple Aim’ by adding a fourth goal: improving the work life and wellbeing of health‐care providers. This change recognises that clinician and staff experience is a prerequisite for achieving better patient outcomes, safer care and sustainable cost reductions. During this period legislative and policy levers to improve wellbeing also emerged. For example, nurse staffing ratio laws (such as California's AB394) drew attention to the connection between staffing levels, nurse workload and both patient and nurse outcomes. Poor work environments correlated with higher nurse burnout, lower job satisfaction and greater intent to leave, whilst adequate staffing helps protect nurse wellbeing by reducing overload, fatigue and in turn improving job satisfaction and retention (Aiken et al. 2008). Our recent monograph argues that nurse staffing, psychological safety, teamwork and willingness to speak up, together with good staff health and psychological wellbeing at work, are essential environmental prerequisites for healthcare improvement and high‐quality care (Maben, Ball, and Edmondson 2023).

In parallel, over the last 20 years, technology and electronic health records began to reshape clinical workflows in some countries. Although intended to improve efficiency, they also created new burdens, increased documentation requirements, frequent alerts and reduced time for direct patient care, which have negatively affected wellbeing and, at times, made the Registered Nurse (RN) role less satisfying. These changes coincided in many countries, with greater reliance on second‐level nurses and assistants, removing RNs from the bedside and increasing their oversight and administrative responsibilities. Some have argued that this shift has sometimes displaced direct patient interactions for RNs, known to be some of the most rewarding aspects of nursing, making care less safe (Leary 2017).

## 3 Links Between Staff Wellbeing and Patient Experiences and Care Quality

The links between staff wellbeing and patient experience and indeed patient outcomes, are now well established (e.g., Boorman 2009; Raleigh et al. 2012; Maben et al. 2012). My work, for the first time, linked staff and patient experience at the individual patient–staff level and demonstrated that staff wellbeing is an antecedent rather than a consequence of patient experience. Thus, systematically enhancing staff wellbeing is not only important in its own right, given employers' duty of care to their employees, but is also critical to maintaining high‐quality patient care and experience. Staff motivation and wellbeing strongly influence patient experience and local work climate, including coworker and supervisor support, a positive organisational culture and low emotional exhaustion, is critical to sustaining staff wellbeing and enabling compassionate, safe and responsive care (Maben et al. 2012).

## 4 Terminology

Terminology has also changed over time. Nurse wellbeing is multifaceted. Encompassing physical health, emotional affect, job satisfaction, work‐life integration and the ability to provide safe, compassionate care. Psychological ill‐health includes stress, anxiety, low mood, together with early causes (such as trauma or moral distress) and consequences over time (burnout, absenteeism, reduced resilience, retention problems) (Taylor et al. 2024). Early 19th‐century discourse on nursing emphasised self‐sacrifice and the earliest research studies focused on concepts such as work stress, anxiety and occupational strain and did not use contemporary terms like ‘wellbeing’ or ‘burnout’ which are now well understood and well established.

Use of the term wellbeing has however become ubiquitous and perhaps as a result has recently been critiqued as performative, giving rise to the term ‘wellbeing washing’, the practice of presenting superficial or performative wellbeing initiatives that reduce support to a tick‐box exercise and leave nurses feeling unsupported (personal communication). Contemporary research frames wellbeing as closely tied to work environment, staffing, leadership, relational job design, organisational culture systemic supports and thriving and flourishing at work, rather than mere survival (West et al. 2020).

## 5 What Can Help: Individual Versus Organisational Interventions and Approaches?

A large body of research has thus documented various elements of the problem and the association between staff psychological wellbeing and patient experience. Ongoing research is developing and testing interventions that help staff deliver high‐quality care while also supporting psychological wellbeing.

For many years there was a focus on offering individuals specific training such as mindfulness and resilience training. Whilst this may at times be helpful, targeting individual coping rather than changing organisational factors like workload, staffing and culture cannot be the only answer (Maben et al. 2024; Stewart et al. 2024). Over time it has been recognised that individual interventions are not enough and there has been something of a backlash about promoting individual resilience whilst staff have no control over or ability to improve workplace conditions. Just before the pandemic we argued that resilience training for nurses cannot be a substitute for safe, well‐resourced working conditions (Maben and Bridges 2020). We cited Traynor (2017) to warn that framing resilience as an individual duty risks blaming staff for problems that arise from systemic issues and it should not be used as a mechanism to shift responsibility away from employers onto individual practitioners. Asking nurses to be ‘resilient enough’ to cope with chronic understaffing, excessive workloads and organisational failures is unacceptable.

In the 1980s and 90s clinical supervision became an important space for nurses to process their work and gain insight from other experienced nurses (Faugier and Butterworth 1994). Whilst the focus was on clinical aspects of caring it also allowed nurses to reflect on their practice and their emotional responses to challenging situations. There were also attempts to do group clinical supervision which may be considered forerunners to Schwartz Rounds (see below).

From 2010 onwards, increasing emphasis was placed on evidence‐based organisational strategies to enhance staff wellbeing, with Magnet approaches evaluated in new settings (Chen and Johantgen 2010; Sermeus et al. 2022) alongside the development of novel interventions and whole new ways of working (e.g., Buurtzorg nursing in the Netherlands). As opportunities for staff to come together and process work‐related challenges diminished, due to increased workload and longer shift times, new approaches were introduced to create dedicated reflective spaces. One example is Schwartz Rounds which were initially developed and evaluated in the United States and subsequently implemented in the United Kingdom, Ireland and Australia. In the US, Lown and Manning (2010) demonstrated that Schwartz Rounds provide a safe, valued multidisciplinary forum in which staff reflect on the emotional and relational dimensions of care, fostering empathy, mutual understanding and commitment to compassionate practice. They also found a dose‐effect, with regular participants experiencing stronger benefits, including greater emotional support, reduced feelings of isolation and improved teamwork, compared to occasional attenders. The first national UK evaluation found that Schwartz Rounds helped staff feel more supported, reduced isolation and increased tolerance, empathy and compassion for self and others, as well as enhancing honesty, openness and teamwork (Maben et al. 2018). Regular attendance was also associated with reduced psychological distress, with GHQ‐12 caseness scores decreasing from approximately 25% to 12% over 8 months, compared with only minimal change in non‐attenders. Typically held monthly, Rounds provide a 1 h, facilitated, non‐hierarchical reflective space in which staff can share (or simply listen to) the social, emotional and ethical challenges of their work, thereby shifting the focus from individual resilience to collective, relational and organisational support.

Recent work examining ‘Care Under Pressure’ among doctors, nurses, midwives and paramedics has used a realist lens to synthesise the literature and highlights the need to shift away from individually focused interventions toward approaches that make workplaces fundamentally healthier through system‐level change and culture improvement (Maben et al. 2024; Melvin et al. 2025). Co‐design methods are emphasised, involving healthcare staff in developing solutions to address wellbeing challenges, alongside recognition that essential needs, such as access to hot food, hydration and rest breaks, must be met and that interventions should be closely aligned with identified problems to support wellbeing and avoid harm (Maben et al. 2024, Melvin et al. 2025). Across these professions, the causes of psychological ill‐health appear more similar than different, suggesting that most interventions need not be profession‐specific; however, particular stressors may be amplified in some groups, such as those with frequent trauma exposure, at vulnerable transitions (such as newly qualified nurses), or working in isolated roles. This body of work also highlights how organisational cultures, rooted in blame rather than learning can undermine staff psychological wellbeing. Bullying and harassment are consistently reported in healthcare, particularly affecting minority staff and women in high‐income countries, although the issue is prevalent globally. This research also identifies persistent tensions in efforts to improve psychological wellbeing, including the challenge of promoting wellbeing within a ‘serve‐and‐sacrifice’ ethos in which organisational priorities override staff needs; and that interventions insufficiently recognise cumulative chronic stressors (Maben, Taylor, et al. 2023; Maben et al. 2024).

**FIGURE 1:**
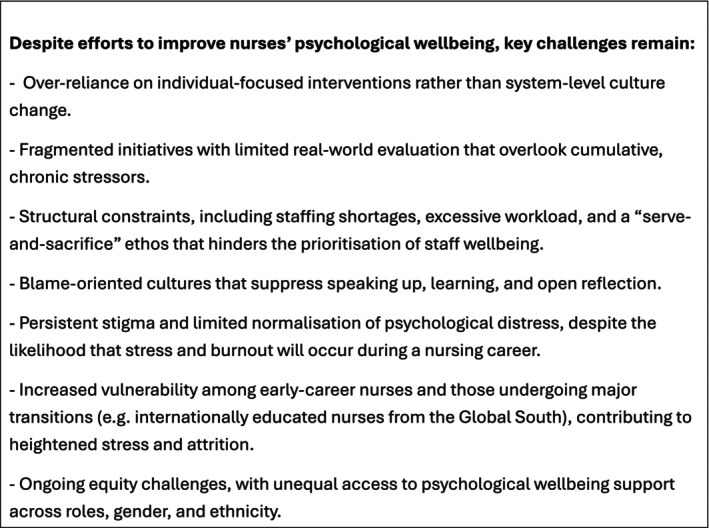
Critical reflections on key challenges (Adapted from Maben et al. 2024).

In summary there are now many interventions to support wellbeing in high‐income countries; some might say too many, making it hard to know which to select or drop and not all are evaluated or take account of context sufficiently. In contrast, many Global South nations face underfunded health systems, compounded by international nurse recruitment drives, limited psychological health support and cultural stigma around emotional vulnerability (Selamu et al. 2017; Abo Shereda et al. 2025), meaning globally there is great disparity and inequity and many remaining challenges (see Figure 1).

## 6 COVID‐19 Pandemic‐ a Significant Shock and Milestone

The pandemic delivered a systemic shock that sharply exposed the psychological toll on nurses, forcing health systems and the public to recognise that supporting nurse psychological wellbeing is not optional, but is essential for sustaining the workforce and ensuring patient safety. The COVID‐19 pandemic exposed and amplified pre‐existing vulnerabilities in nurse wellbeing. Although some rapid innovations emerged (Maben et al. 2024), for example, Wobble Rooms and WhatsApp groups for redeployed staff, these were modest and typically unevaluated, ad hoc responses that mainly provided opportunities for mutual support rather than sustainable solutions. Our research (Maben et al. 2024) found that the pandemic intensified existing workplace stressors, including stress and trauma, moral distress and high emotional labour and reinforced a prevailing ‘serve‐and‐sacrifice’ ethos, placing even greater pressure and strain on nurses, midwives and paramedics.

There was notable investment in some high‐income countries, including the UK, USA, Canada and Australia. In the UK, NHS England and NHS Improvement announced an additional £15 million in October 2020 to strengthen mental‐health support for staff, including nurses and support staff (NHS England 2020). However, much of this support took the form of online apps and digital resources, which many nurses reported were difficult to access or ill‐suited to their needs; when at work, they had little time to use them and when at home, they preferred to detach from work‐related content. Globally, the World Health Organization and the International Council of Nurses called for increased investment, particularly in lower‐income settings, yet there appears to have been very little, if any, additional resourcing specifically directed toward nurse psychological wellbeing in these countries during the pandemic (WHO 2022a, 2022b; ICN 2023).

What we do know is that the pandemic had a considerable impact on nurses' psychological wellbeing, with many reporting increased anxiety, stress and, in some cases, symptoms consistent with PTSD. Where possible, especially in high‐income countries, some nurses stepped back from frontline roles through early retirement or by moving to different areas of practice, away from the settings where they worked during the pandemic. Many described themselves as ‘forever altered’ (Maben et al. 2022; Maben, Conolly, et al. 2023) suggesting recovery will be prolonged, yet there has been organisational and cultural pressure to move on rather than to examine or address the lasting effects of the pandemic (Maben et al. 2022; Maben, Conolly, et al. 2023).

## 7 Consolidation and Future Directions

Nursing is a highly flexible profession that offers many pathways, diverse specialties, varied settings and careers in clinical practice or research and academia. Despite well‐documented stresses and challenges, a substantial body of work identifies what keeps nurses in their roles. Key intrinsic motivators include the ability to help others, to deliver high‐quality person‐centred care, the variety of the work and a strong sense of vocation, professional identity and meaning. Important organisational buffers include an environment that enables meaningful person‐centred care, supportive colleagues and teamwork, recognition and feeling valued, manageable workloads, professional autonomy and visible leadership support. Indeed, recent doctoral research indicates that professional enjoyment in nursing is closely tied to identity as a nurse, making a difference in patients' lives, feeling valued and having the nursing voice heard (e.g., Loft and Lomholt 2020; Donohue 2023).

Research consistently shows that nurses derive deep meaning from relationships with patients and colleagues and feel most satisfied when they make a tangible difference to patients, families and carers. This sense of purpose is a central reason many people enter and remain in the profession. The challenge for the mid‐21st century is ensuring that health systems and workplaces preserve and strengthen those sources of meaning so nurses can continue to deliver person‐centred care, experience professional fulfilment and sustain long careers.

## 8 The Future of Nursing is AI?

Artificial intelligence (AI) has been suggested as an important tool for nurses of the future, to augment nursing practice by reducing routine burdens, improving decision making and freeing nurses to spend more time on direct patient care. AI‐enabled social or companion robots, predictive models and conversational assistants such as ChatGPT, may further enhance the delivery and management of nursing care. One of AI's major benefits is its clinical decision support capabilities, as AI‐based tools provide real‐time, evidence‐based insights, allowing nurses to make more informed and well‐founded decisions (Lin et al. 2024). Interestingly there are reports of AI being used to measure nurse burnout and some reports of predictive AI models that detect patterns of exhaustion among nurses, allowing for preventive interventions (Pereira et al. 2025).

Indeed, many of these things are already taking place, with some positive measurable but also mixed benefits for nursing practice. Evaluated applications have demonstrated time savings for routine documentation and earlier detection of patient deterioration, improvements in some safety process metrics and positive nurse perceptions when tools are well integrated and co‐designed with frontline staff (Hicks et al. 2024; Jacques et al. 2025; Canfell et al. 2024). However, qualitative evidence, reveals significant tensions (documentation burden, workflow disruption, variable acceptance) (Canfell et al. 2024). Systematic reviews have also shown that impacts on clinical outcomes and sustained reductions in workload are inconsistent. Benefits depend on how well the tool integrates with care delivery and implementation strategies, while bias, poor integration and lack of governance can offset gains. Digitalisation for example, does not simply reduce workload; it changes it, often adding new demands (e.g., digital counselling, messaging) requiring new skills (Kaihlanen et al. 2023; Schlicht et al. 2025). Research calls for co‐design with nurses and investment in training and support (Pereira et al. 2025; Ronquillo et al. 2021).

But what of companion care robots in the future? Given patients' need to connect, be heard and receive empathy, the nursing role will remain essential and cannot be wholly replaced by AI or robots. In some settings care robots may provide responsive companionship and take on routine tasks, potentially freeing nurses to spend more time on assessment, complex decision‐making and other important aspects of practice. However, direct bedside care is a primary source of professional reward and job satisfaction; if robots push RNs further away from patients, they are unlikely to improve psychological well‐being or retention and, when poorly implemented, may introduce new burdens for staff. For example, additional tasks such as charging, cleaning, transporting and minor repairs of robotic devices can be time‐consuming and may increase nursing workload, with everyday technical maintenance often falling to nursing staff and creating unanticipated burdens that offset some of the expected relief from automation (Servaty et al. 2020; Adeyemo et al. 2025).

## 9 Future of Interventions to Support Psychological Wellbeing

It is fair to say that despite decades of strong research and a much better understanding of the challenges nurses face and the causes of poor psychological wellbeing at work, the overall picture has not substantially improved. Rates of stress and burnout have remained high in many countries and the interventions that exist have largely not changed the situation substantially‐ with a few notable exceptions. Interventions remain fragmented and too often target individuals and the challenges in Figure 1 remain. What is needed is a broader focus on the elements of practice, management and culture that make work meaningful and allow nurses to thrive. That means addressing the everyday drains on nurses' time and morale, unreliable equipment and IT, inadequate staffing, a sense of not being heard and barriers to speaking up about necessary change. Nurse voices must be heard loudly and clearly: they know what will keep them in the workforce and need to be listened to. Fundamental culture and system change is essential.

Today, nursing has far greater visibility of psychological wellbeing as a legitimate professional concern than it did 50 years ago and that progress is worth celebrating. Education programmes increasingly include reflective practice, self‐care strategies, teamwork and emotional wellbeing. Nurses are more likely to expect organisational support, yet role expectations have expanded (advanced practice, leadership, research) which offers professional growth but may also require new coping capacities.

**FIGURE 2:**
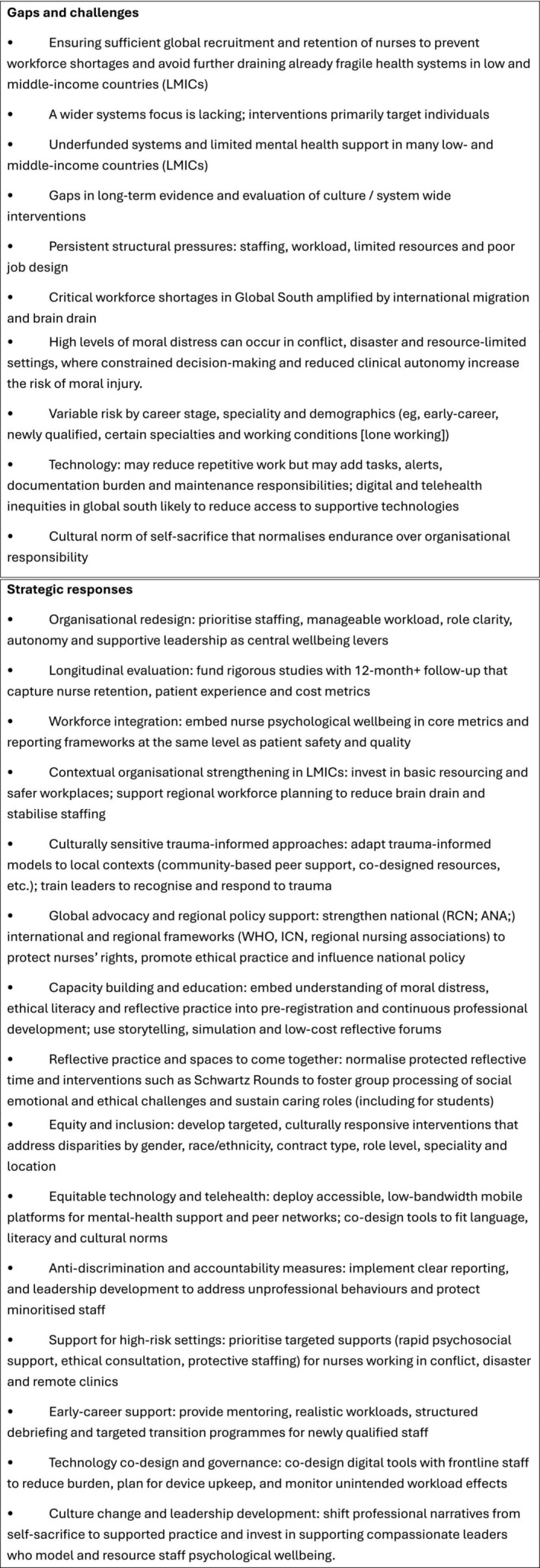
Critical gaps and challenges and strategic responses for nurse psychological wellbeing.

There may also be greater challenges ahead (see Figure 2). The global shortage of nurses could persist, leaving fewer RNs available to provide direct patient care and to supervise support staff, with potential negative effects on care quality (Buchan 2002, 2008; Leary et al. 2024). Global pressures, falling birthrates in many countries, climate change, conflict, population displacement, migration and the rising burden of disease and ageing populations, will intensify strain on health systems and on nurses. The costs of poor wellbeing, turnover, absenteeism and reliance on agency staff are already well documented, so embedding nurse wellbeing into every workforce strategy is essential for sustainable healthcare systems. Effective approaches must address organisational and cultural drivers, not only individual resilience (Maben and Bridges 2020; Maben et al. 2024). Technology, new staffing models, strong education pipelines and better support for students and organisational culture initiatives are seen as levers for change, but as discussed are not without their detractors. However, the COVID‐19 shock may have helpfully emphasised that nurse psychological wellbeing is essential for system resilience and capacity.

## 10 Conclusion

Over 50 years, nursing has shifted from valuing sacrifice to recognising nurse psychological wellbeing as central to professional practice. Indeed, the past 50 years have seen remarkable progress in recognising, researching and addressing nurse psychological wellbeing. From the early measurement of burnout to the system‐wide shocks of a global pandemic, the nursing profession has been forced to rethink how it supports its workforce. The link between nurse wellbeing, patient care and outcomes and health system performance is now widely accepted. However, significant challenges remain, especially around global issues, structural redesign and how to intervene at a systems, rather than individual level, as well as evidence on the longevity of interventions, support for early‐career nurses, equity and the integration of technology. This commentary has highlighted key milestones in research, policy, technology and education, to map how nurse psychological wellbeing has evolved, the ways it has shaped nursing practice and patient care and where the gaps and future challenges and opportunities lie.

Going forward, the nursing profession and healthcare systems must not only ask ‘how do we help nurses cope?’ but ‘how do we design systems and jobs where nurses can thrive and flourish?’ True sustainability in nursing depends on far more than resilience training; it requires adequate staffing, professional autonomy, fairness, meaningful work, a culture of learning, support and openness, well‐designed organisational interventions and thoughtful use of AI and technology to ensure that nurses continue to find meaning in their work. Nurse psychological wellbeing must become central to organisational values and policies and be elevated to the same status as patient safety, clinical quality and workforce planning.

The past 50 years have seen a profound shift in how health care systems across the globe value and support their nursing workforce, not just as caregivers, but as human beings navigating trauma, ethical and patient complexity and interpersonal challenges. The well‐being of nursing staff is a global imperative. But everything is not yet equal; while high‐income countries have made strides in addressing trauma, moral distress and unprofessional behaviours, global majority nurses often navigate these challenges with fewer resources and greater systemic barriers. A truly equitable future demands international solidarity, culturally grounded interventions and a serious global commitment to healing the healers, wherever they work.

Thus, as we look ahead, the profession must continue to evolve by embedding support for nurse psychological wellbeing into the very fabric of healthcare. Only then can nurses truly care well, not in spite of their distress, but through systems that honour and support their humanity.

## Funding

The author has nothing to report.

## Conflicts of Interest

The author declares no conflicts of interest.

## Data Availability

Data sharing not applicable to this article as no datasets were generated or analysed during the current study.
